# Asessment of ageing effect on plantar tissue stiffness

**DOI:** 10.1186/1757-1146-5-S1-P27

**Published:** 2012-04-10

**Authors:** Jee-Chin Teoh, Wen-Ming Chen, Taeyong Lee

**Affiliations:** 1 Division of Bioengineering, National University of Singapore, Singapore

## Background

Foot abnormality has become a public health concern. Early detection of pathological soft tissue is hence an important preventive measure, especially to the elderly who generally have a higher risk of foot pathology (i.e. ulceration) [1]. Accumulated changes over time diminish the mechanical properties of plantar soft tissue, causing easy breakdown of tissue and instability of foot during walking. Non invasive in-vivo assessment on plantar soft tissue mechanical responses is hence needed. This is to identify abnormal soft tissue such that early precaution measures can be taken to avoid foot pathology that requires long healing period.

The purpose of this study is to assess ageing effect on plantar tissue using an improved version of instrumented in vivo tissue tester [2]. It also aims to provide a useful parameter to identify tissue with high ulceration risk. This is done by varying metatarsophalangeal (MTP) joint configurations and imposing large tissue deformation to the soft tissue.

## Methods

**Figure 1:**
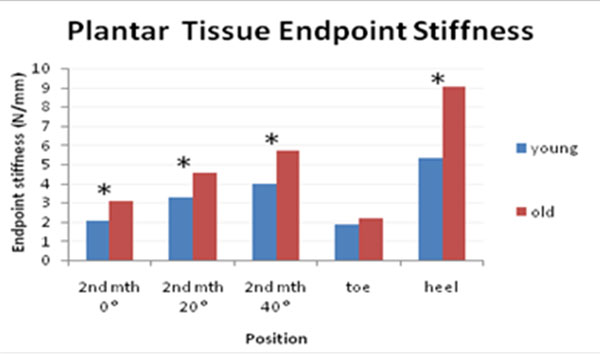
Comparison of soft tissue stiffness between young and old subjects

10 young (20-30 years) and 10 old subjects (60-70 years) participated. During the testing, the indentor tip probed the metatarsal head (MTH) pad tissue at 3 different dorsiflexion angles of 0°, 20°, 40° as average MTP dorsiflexion was 25°-47° during walking[3]. Maximum tissue deformation was set at 5.6mm (close to literature data). [4] Experiment was repeated on 1^st^ hallux and heel. Tissue stiffness obtained from tissue response curve was compared (Figure 1).

## Results

As MTP dorflexion increased, old subjects had a steeper increase in stiffness value as compared to the young. Old subjects also showed significantly higher tissue stiffness in 2^nd^ MTH and heel region.

## Conclusion

Notably, aging resulted in stiffer tissue property. Ageing effect was the most prominent as the MTP dorsiflexion was maximum. This critical scenario was of utter importance as it had highest ulceration risk. Previous work had failed to consider MTP dorsiflexion and large tissue deformation leading to a less critical and less useful stiffness measurements. This study successfully demonstrated the positive relationship aging and soft tissue stiffness in a realistic manner by better replicating actual gait condition. It also provided a more useful stiffness values in identification of potentially abnormal soft tissue.
